# Metabolic engineering of *Bacillus megaterium* for heparosan biosynthesis using *Pasteurella multocida* heparosan synthase, *PmHS2*

**DOI:** 10.1186/s12934-019-1187-9

**Published:** 2019-08-12

**Authors:** Asher Williams, Kamil S. Gedeon, Deepika Vaidyanathan, Yanlei Yu, Cynthia H. Collins, Jonathan S. Dordick, Robert J. Linhardt, Mattheos A. G. Koffas

**Affiliations:** 1 Department of Chemical and Biological Engineering, Rensselaer Polytechnic Institute, Troy, NY 12180 USA; 2 Department of Chemistry and Chemical Biology, Rensselaer Polytechnic Institute, Troy, NY 12180 USA; 3 Department of Biology, Rensselaer Polytechnic Institute, Troy, NY 12180 USA; 4 Center for Biotechnology and Interdisciplinary Studies, Rensselaer Polytechnic Institute, Troy, NY USA

**Keywords:** Heparosan, Glycosaminoglycans, *Bacillus megaterium*, Heparosan synthase, Metabolic engineering

## Abstract

**Background:**

Heparosan is the unsulfated precursor of heparin and heparan sulfate and its synthesis is typically the first step in the production of bioengineered heparin. In addition to its utility as the starting material for this important anticoagulant and anti-inflammatory drug, heparosan is a versatile compound that possesses suitable chemical and physical properties for making a variety of high-quality tissue engineering biomaterials, gels and scaffolds, as well as serving as a drug delivery vehicle. The selected production host was the Gram-positive bacterium *Bacillus megaterium*, which represents an increasingly used choice for high-yield production of intra- and extracellular biomolecules for scientific and industrial applications.

**Results:**

We have engineered the metabolism of *B. megaterium* to produce heparosan, using a T7 RNA polymerase (T7 RNAP) expression system. This system, which allows tightly regulated and efficient induction of genes of interest, has been co-opted for control of *Pasteurella multocida* heparosan synthase (*PmHS2*). Specifically, we show that *B. megaterium* MS941 cells co-transformed with pT7-RNAP and pPT7_PmHS2 plasmids are capable of producing heparosan upon induction with xylose, providing an alternate, safe source of heparosan. Productivities of ~ 250 mg/L of heparosan in shake flasks and ~ 2.74 g/L in fed-batch cultivation were reached. The polydisperse *Pasteurella* heparosan synthase products from *B. megaterium* primarily consisted of a relatively high molecular weight (MW) heparosan (~ 200–300 kD) that may be appropriate for producing certain biomaterials; while the less abundant lower MW heparosan fractions (~ 10–40 kD) can be a suitable starting material for heparin synthesis.

**Conclusion:**

We have successfully engineered an asporogenic and non-pathogenic *B. megaterium* host strain to produce heparosan for various applications, through a combination of genetic manipulation and growth optimization strategies. The heparosan products from *B. megaterium* display a different range of MW products than traditional *E. coli* K5 products, diversifying its potential applications and facilitating increased product utility.

**Electronic supplementary material:**

The online version of this article (10.1186/s12934-019-1187-9) contains supplementary material, which is available to authorized users.

## Background

### Heparosan structure and function

**Fig. 1:**
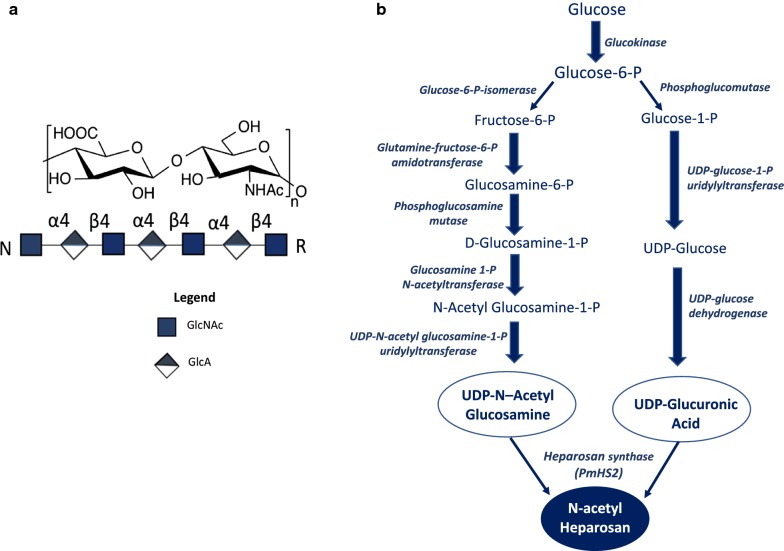
**a** The chemical structure and symbolic representation of the disaccharide repeating unit of heparosan. N: non-reducing end, R: reducing end. **b** Metabolic pathway showing the biosynthesis of heparosan from relevant precursors. Enzyme names are italicized

Heparosan, a member of the glycosaminoglycan (GAG) family, is comprised of [→4) β-d-glucuronic acid (GlcA) (1→4) *N*-acetyl-α-d-glucosamine (GlcNAc) (1→)]_n_ repeating disaccharide units [1] as shown in Fig. 1a. This polysaccharide is the natural precursor of heparan sulfate (HS), as well as heparin, a widely used drug [2, 3] that is primarily employed in surgery to stop vein thrombosis and also administrated in other medical procedures [4]. Heparosan is composed of the same two monosaccharide component sugars as hyaluronan but differing glycosidic bonds (the β1,3-bond between glucuronic acid and *N*-acetyl-glucosamine in hyaluronan is replaced by an α1,4-bond in heparosan) [5]. Heparosan’s unique properties make it ideal for producing certain biomaterials and viscoelastics [6]. Additionally, since stretches of heparosan exist in the HS chains found on nearly every human cell, it is expected to be biocompatible in the human body, making it a suitable drug delivery vehicle [7].

### Genes associated with the biosynthesis of heparosan and its precursors

Bacterial capsules composed of heparosan have been reported in *Escherichia coli* K5 [8] and *Pasteurella multocida* Type D [9]. The biosynthesis of heparosan (Fig. 1b) is regulated in *E. coli* K5 by four genes, *kfiA*, *kfiB*, *kfiC*, and *kfiD*. *KfiD* encodes for a UDP glucose dehydrogenase and the role of the protein encoded by *kfiB* is unclear [10]. Both *kfiA* and *kfiC* are required for polymerization activity, since they are mono-action transferases, respectively encoding for *N*-acetylglucosaminyltransferase and D-glucuronyltransferase [10, 11].

In contrast to what is observed in *E. coli*, in *P. multocida* Type D, the synthesis of heparosan is performed by only one enzyme with two glycosyltransferase activities—the heparosan synthase PmHS1. When the *P. multocida* Type D heparosan synthase gene *pmhssA* was cloned to express PmHS1 active proteins [9], a cryptic gene *pmhssB* encoding for an active recombinant heparosan synthase PmHS2 was discovered in *P. multocida* Type A, D, and F, based on homology with *pmhssA* [9, 12]. These synthases (PmHS1 and PmHS2) are both dual action glycosyltransferases exhibiting glucuronyl transferase and *N*-acetylglucosaminyl transferase activities. PmHS2 differs from PmHS1 in the MW distribution of synthesized heparosan polymers as well as its ability to create novel GAG polymers from unnatural donor sugar analogs [13–15].

While *E. coli* K5 typically produces heparosan in the range of 50–80 kD [1, 16], the *Pasteurella* heparosan synthases produce various sizes of monodisperse high molecular weight heparosan [14]. Since polymer size distribution affects properties like viscosity, chain entanglement and solubility, the relatively high MW heparosan produced in this study will be more suitable than *E. coli* K5 heparosan for producing biomaterials like hydrogels and viscoelastics [6]; while the smaller sized fractions could serve as heparin precursors. Compared to its homolog PmHS1, the PmHS2 glycosyltransferase protein is better able to generate polysaccharides through de novo synthesis [17] and has been shown to be more flexible in its ability to polymerize various C2 HexNAc sugar analogs [5]. PmHS2 also possesses increased donor and acceptor flexibility, possibly resulting in a more dynamic survival response for microbes under stressed conditions or environmental signals like relative UDP-sugar availability [5].

### Metabolic engineering strategies for heparosan production

Harnessing engineered microorganisms for the production of heparosan and other compounds has attracted a great deal of interest, as chemical synthesis is often time-consuming and costly [18], and the traditional animal-extraction route has issues related to product variability, contamination risks and limited availability of source tissues [19]. Several microorganisms have been exploited for heparosan production, either through engineering native heparosan-producing strains to improve yields or by transferring product-specific enzymes or complete metabolic pathways to a more genetically tractable microorganism [20].

### *Bacillus megaterium* as an expression system

*Bacillus megaterium* has been increasingly used as a host for the production of heterologous genes since it lacks alkaline proteases and has been found to efficiently express and secrete foreign proteins [21, 22]. The plasmidless strain DSM319, parent strain of the protease deficient MS941 strain used in this study, is well-known industrially [22–24], and better characterized genetically and physiologically than most other bacilli [22]. Importantly, unlike *B. subtilis,* this host strain is also asporogenic on common media, ensuring that vegetative cells will not forgo normal cellular division to form endospores in stressful growth conditions [25]. A limited number of strong inducible promoter systems are available for *B. megaterium*, including sucrose-inducible [26] and xylose-inducible promoters [27]. The most prominent *B. megaterium* expression system is based on the RNA polymerase of the T7 bacteriophage (T7 RNAP), originally developed for *E. coli* [28], with the T7 RNA polymerase gene under the control of the *xylA* promoter. This system is based on two compatible plasmids: pT7-RNAP and pPT7 [29].

The broad assortment of genetically characterized strains, genetic methods, vectors, and genomic sequences make *B. megaterium* an attractive organism for industrial and experimental applications [30]. Additionally, the stable maintenance of two freely replicating plasmids makes the *B. megaterium* T7 RNAP-driven expression system a suitable alternative to the widely used *E. coli* system. With a cell length of up to 4 µm and a diameter of 1.5 µm, *B. megaterium* is amongst the biggest known bacteria, possibly giving it the potential for higher product yields [31]. These features make *B. megaterium* an ideal host for generating the unsulfated GAG heparosan, for the first time using the bifunctional *PmHS2* gene, facilitating the production of polysaccharides with a unique range of molecular weights for varied applications.

## Results

### Recombinant *B. megaterium* strains produced up to 250 mg/L in shake flask experiments

**Fig. 2:**
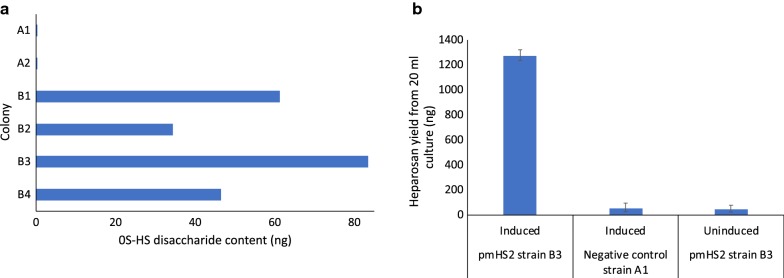
Screening of *B. megaterium* colonies. **a** Various colonies from successful *B. megaterium* transformations screened for heparosan production, where 0S-HS refers to the unsulfated heparosan disaccharide units as quantified by LCMS analysis. A1, A2: colonies of negative control strain harboring pPT7_X plasmid; B1, B2, B3, B4: colonies harboring the pPT7_PmHS2 plasmid. **b** Heparosan titers from shake flask control experiment of the pPT7_PmHS2 strain (B3) induced and uninduced, and pPT7_X strain (A1) induced

Colonies were obtained from successful co-transformation of the pPT7_PmHS2 and pT7-RNAP constructs into *B. megaterium* MS941. pPT7_PmHS2 is responsible for the T7 RNAP-dependent expression of the heparosan synthase gene, and the pT7-RNAP construct contains the T7 RNA polymerase gene under control of the strong *xylA* promoter. The negative control strain contained the pT7-RNAP plasmid, along with a pPT7_X construct that harbored a different gene. The colonies were screened by overnight growth with the appropriate antibiotics and four colonies were obtained (Fig. 2) for pPT7_PmHS2 (B1–B4) and two for the negative control pPT7_X (A1 and A2). The better-producing colonies were larger and displayed a more circular morphology, compared to poorer producers which formed small and irregularly shaped colonies.

LCMS disaccharide analysis of heparosan produced in the supernatant of the selected colonies indicated the presence of a single disaccharide with mass-to-charge ratio (*m/z*) of 572, corresponding to uronic acid/*N*-acetyl hexosamine and consistent with the uniform repeating structure of heparosan: [→4) β-d-glucuronic acid (GlcA) (1→4) *N*-acetyl-α-d-glucosamine (GlcNAc) (1→)]_n_. Figure 2a illustrates the varying production levels of different colonies and the best producing colony (B3), was selected for further analysis. A control experiment was run with 20 mL shake flasks of the pPT7_PmHS2 strain B3 induced and uninduced, and pPT7_X strain induced. As shown in Fig. 2b, substantial heparosan production was only detected for induced B3, indicating that heparosan was produced due to induction of the *PmHS2* gene. Heparosan was quantified using a standard curve generated by LCMS disaccharide analysis (Additional file 1: Figure S1).

**Fig. 3:**
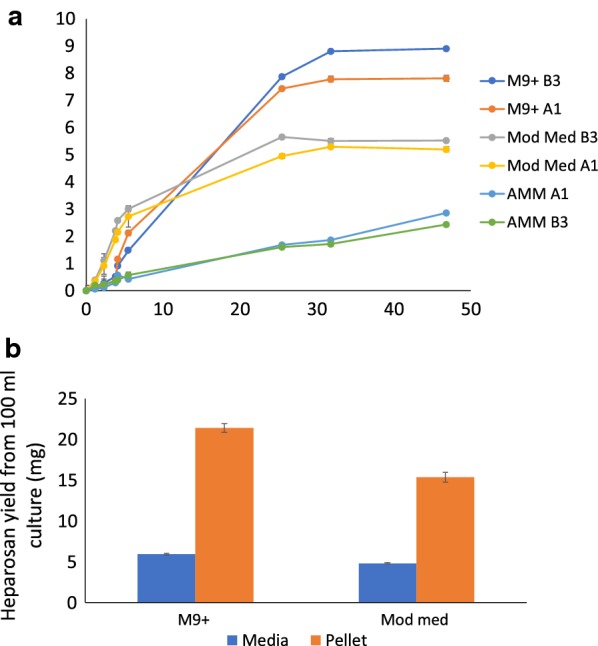
Shake flask growth data. **a** Shake flask growth curves for *B. megaterium* strains grown in modified medium (Mod Med), a rich defined medium from adapted protocols (AMM), and a minimal media optimized for *B. megaterium* growth (M9+). A1 is a negative control strain harboring the pPT7_X plasmid and B3 is a strain harboring the pPT7_ PmHS2 plasmid. **b** Heparosan yields from PmHS2_pPT7 strain grown in M9+ and modified medium (Mod med)

*Bacillus megaterium* cell growth in three media types was tested, with carbon sources of sucrose (modified medium [32]) or glucose (M9+ and AMM), to determine which was best for growth and heparosan yield. The growth curves in Fig. 3a show that the best growth and highest OD_600_ of ~ 9.0 was achieved in M9+ medium. Further analysis of the products from the M9+ and modified medium, where better growth was observed, showed that a higher heparosan yield was achieved with M9+ (Fig. 3b). This minimal media condition is advantageous for our system as it allows the carbon source to be carefully defined for optimized cell growth [33] and also eliminates complex media components associated with modified medium, facilitating simplified product purification from the supernatant [1, 34].

Shake flask cell growth was stopped after ~ 48 h since it was observed that the OD_600_ values gradually decreased when cells entered late stationary phase, accompanied by a moderate degree of cell lysis and acetate buildup in the growth medium. Although this has the potential to increase the availability of heparosan in the supernatant, product purity remained an issue for the cell culture supernatant CPS compared to the cell pellet product. Further optimization of induction conditions showed that the highest yield was achieved when gene expression was induced at OD_600_ values between 0.33 and 0.50 at 37 °C, over a period of 48 h. A summary of all the conditions that were optimized to achieve a maximum titer of ~ 250 mg/L in shake flasks is shown in Additional file 1: Figure S2.

### Recombinant *B. megaterium* strains produced up to 2.74 g/L in bioreactor experiments

**Fig. 4:**
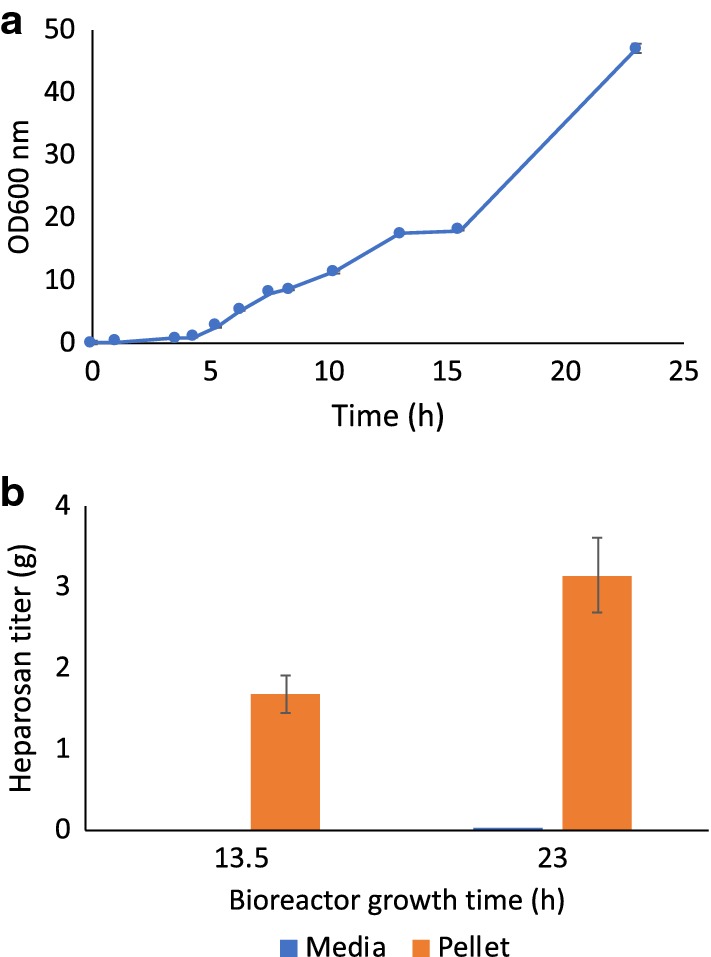
Bioreactor growth data. **a** Bioreactor growth curve for heparosan-producing *B. megaterium* strains grown in (M9+). **b** Bioreactor heparosan titers as quantified by LCMS disaccharide analysis

The heparosan production level was scaled up from shake flasks to a 1.5 L benchtop fermenter using M9+ medium. Optimization of bioreactor growth conditions and feeding strategy has the potential for several fold increase in heparosan yield, as a fed-batch process provides a feed medium that prevents depletion of nutrients and sustains the production phase [34]. A glucose consumption profile was generated over a 24 h period in order to develop an optimized carbon-feeding scheme (Additional file 1: Figure S3). The maximum OD_600_ achieved in the bioreactor when the full capacity of 1.3 L was reached was ~ 47 (Fig. 4a). Heparosan titers increased from ~ 1.2 g/L after 13 h to 2.74 g/L at the end of fermentation, as quantified by disaccharide analysis after heparinase treatment and fluorescent labeling (Fig. 4b). Based on glucose consumption, the bioreactor yield was ~ 17.9 mg heparosan/g glucose, as compared to 12.5 mg heparosan/g glucose for shake flask growth. LCMS analysis also showed that practically all of the CPS remained in the cell pellet as there was no detectable heparosan disaccharide present in the supernatant of the fermentation broth after a ~ 23 h growth period (Fig. 4b).

### Quantification and structural verification of heparosan products by LCMS disaccharide analysis

**Fig. 5:**
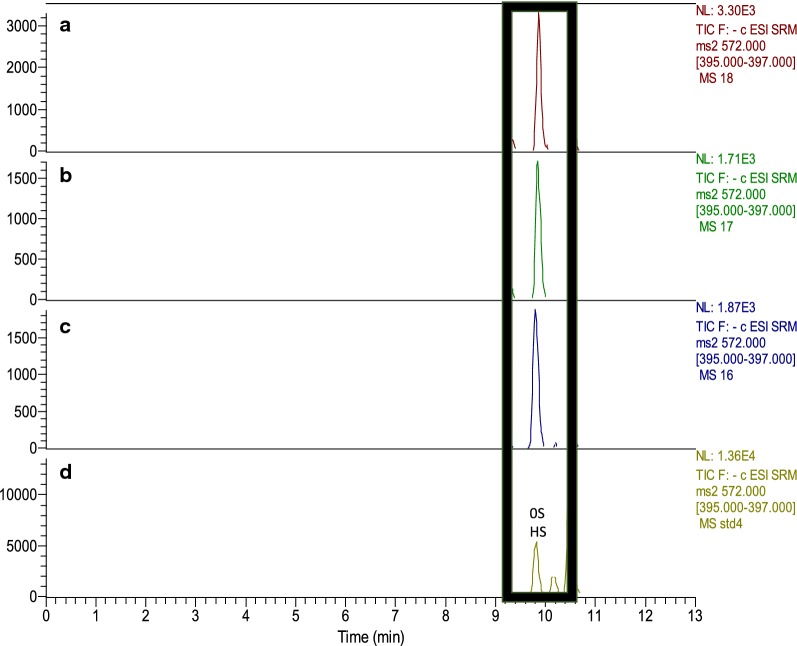
Chromatograms from LCMS/MS MRM analysis of *B. megaterium* heparosan products. **a** Bioreactor cell pellet product, **b** shake flask cell pellet product, **c** shake flask cell culture supernatant product, **d** heparosan disaccharide standard with labeled 0S HS peak

LCMS analysis of the 2-aminoacridine (AMAC)-labeled heparosan products showed that ~ 82% of the shake flask product was found in the cell pellet and the remainder in the cell culture supernatant (Fig. 3b), while all of the bioreactor product was found in the cell pellet only (Fig. 4b). The disaccharide products obtained after heparin lyase digestion were consistent with the uniform repeating structure of an unsulfated heparosan disaccharide standard, with an identical retention time and characteristic *m/z* ratio of 572 as shown in Fig. 5. LCMS disaccharide analysis provides a structure-specific assay for heparosan quantification, compared to colorimetric assays like carbazole [35] where the quantification of GAGs originating from bacterial fermentation is restricted by interference from cellular remains and the growth medium [36]. While liquid chromatography separates mixtures with multiple components, mass spectrometry provides the structural identity of individual components with a high level of molecular specificity and detection sensitivity [37].

### Structural analysis of heparosan products using ^1^H nuclear magnetic resonance (NMR)

**Fig. 6:**
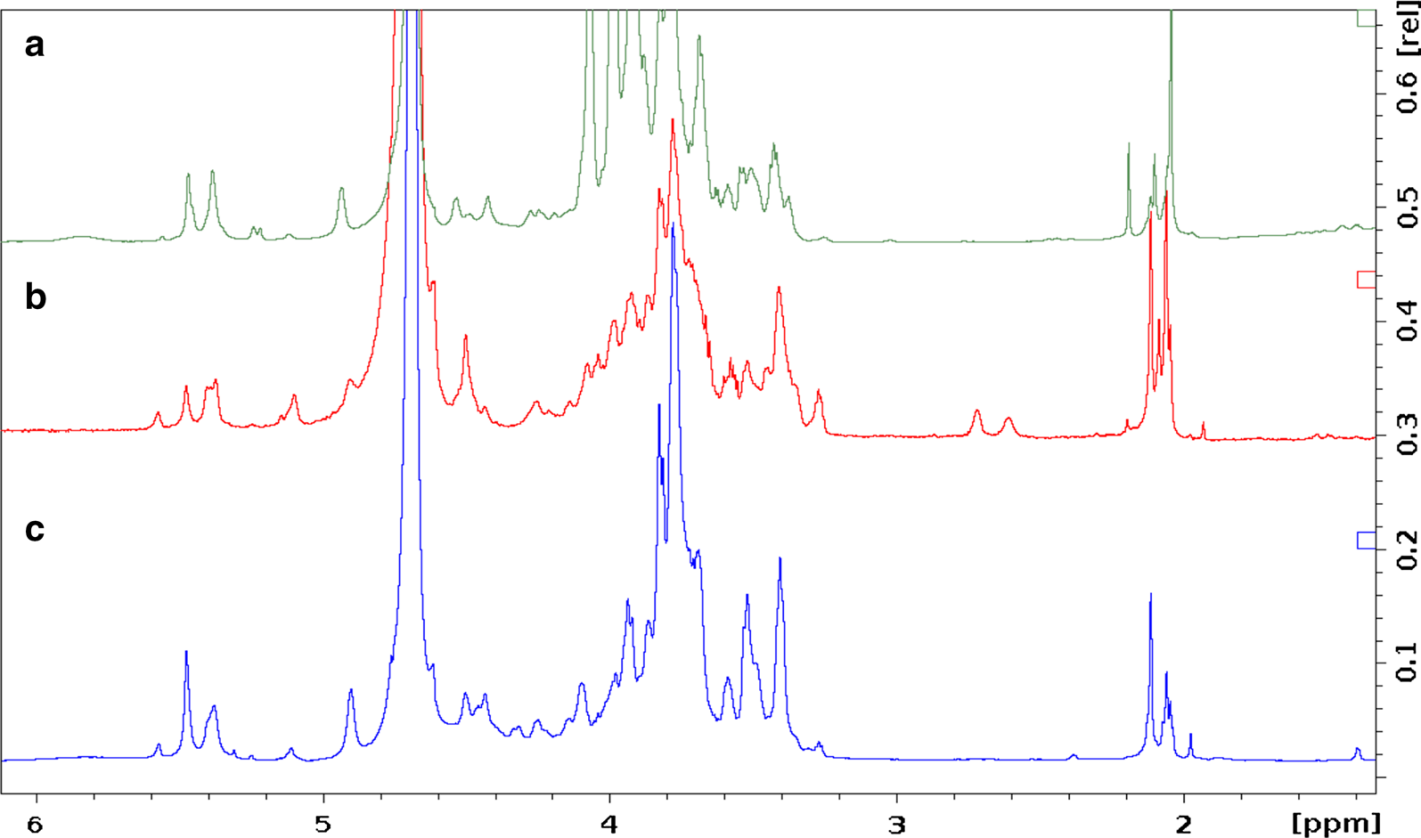
^1^H NMR spectra of heparosan products from the engineered *B. megaterium* strain. **a** Shake flask cell culture supernatant, **b** bioreactor cell pellet product **c** shake flask cell pellet product. The peak assignments are outlined in Table 1

Proton nuclear magnetic resonance (^1^H NMR) data was also acquired for the bacterially produced heparosan products to corroborate the LCMS structural data. This one-dimensional technique is based on highly predictable chemical shifts for specific molecular environments and has been used extensively to elucidate carbohydrate structures [38, 39]. Chemical shifts for the characteristic heparosan peaks shown in Fig. 6 are outlined in Table 1. These characteristic peaks were not present in the spectral data for the negative control strain, pPT7_X.

**Table 1:** ^1^H NMR chemical shift assignments for heparosan products from the engineered *B. megaterium* strain

Proton	Chemical shift (ppm)
GlcNAc, CH_3_	2.05
GlcA H_2_	3.27
GlcA H_3_, H_4_ and GlcNAc H_4_	3.48–3.53
GlcA, H_5_	3.68
GlcNAc H_2_, H_3_, H_5_, H_6_	3.80–3.84
GlcA H_1_	4.62
GlcNAc, H_1_	5.57

Similar to our *B. megaterium* heparosan product, a much lower *N*-acetyl peak at ~ 2 ppm was observed in heparosan produced in *B. subtilis* [32], compared to heparosan from *E. coli* K5, where the *N*-acetyl peak is typically the highest in the ^1^H NMR spectrum (Additional file 1: Figure S4). Studies have shown that the ratio of peak heights varies based on production host and strain, possibly due to differences in metabolic pathway enzymes and the rich diversity of biological contexts in which CPSs are found [38]. Additionally, the NMR spectra of carbohydrates are often relatively difficult to interpret due to a combination of structural diversity at several levels and limited chemical shift dispersion [39, 40].

### *B. megaterium* heparosan products display two distinct molecular weight ranges

**Fig. 7:**
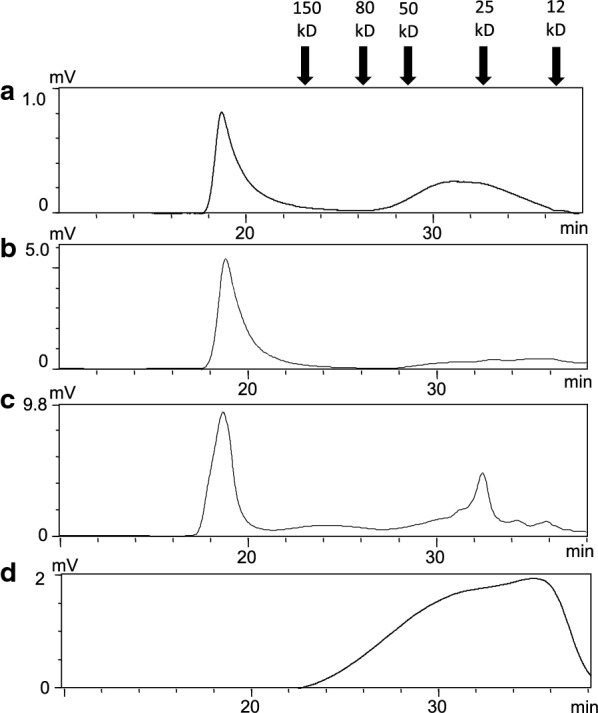
Molecular weight profiles of various heparosan products measured by GPC-HPLC. **a** Bioreactor cell pellet product; **b** shake flask cell pellet product; **c** shake flask supernatant product; **d** *E. coli* K5 heparosan. Molecular weights were determined using dextran calibration standards as indicated by the labeled arrows

**Fig. 8:**
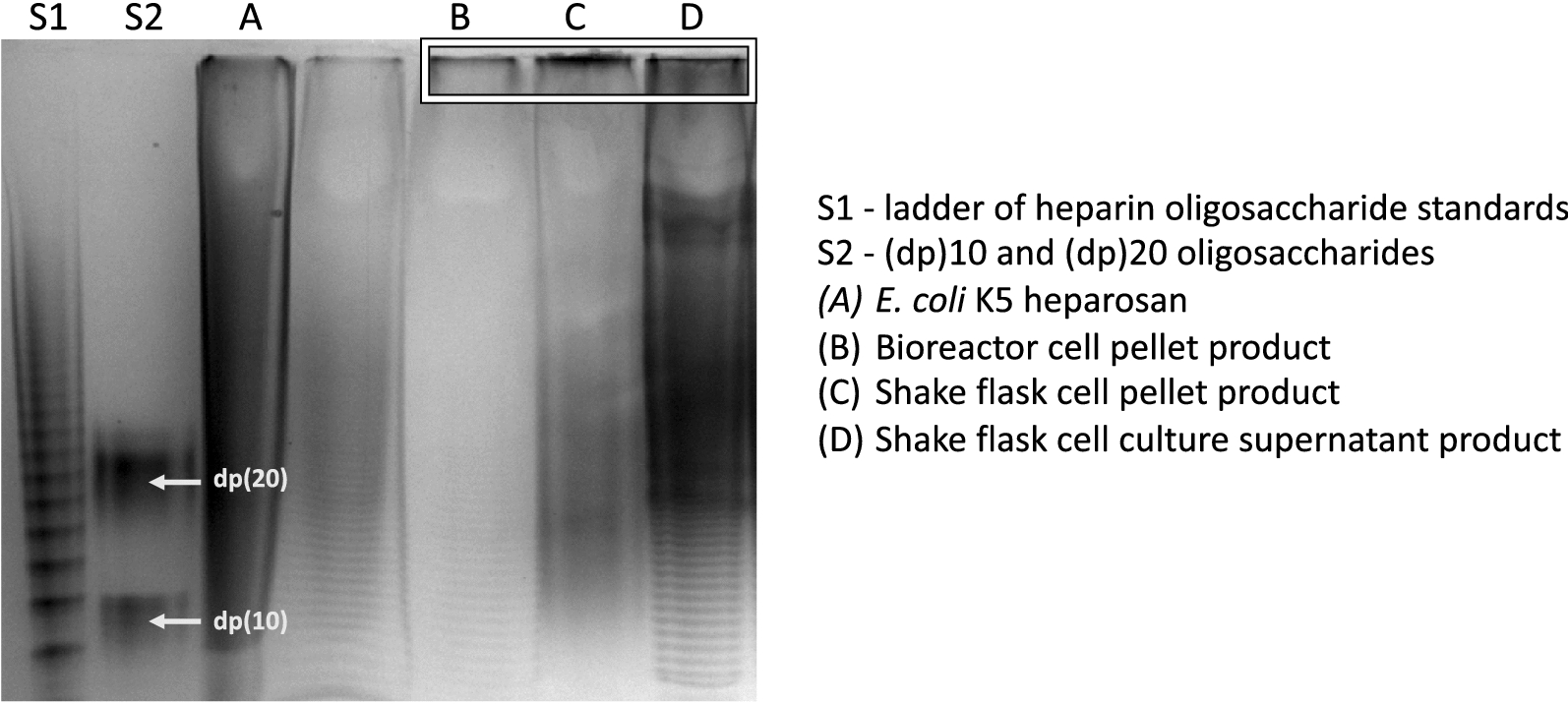
PAGE analysis using 15% resolving gel analysis of various *B. megaterium* heparosan products. S1 shows a ladder of heparin oligosaccharide standards of known molecular weights that were enzymatically prepared from bovine lung heparin [49]. S2 shows the band sizes using structurally defined oligosaccharides of degree of polymerization (dp)10 and (dp)20 [55]. Other lanes show the heparosan products, with the higher MW products in lanes B–D highlighted

Gel permeation chromatography–high performance liquid chromatography (GPC–HPLC) was used to measure the relative molecular mass properties of heparosan products, with dextran (Additional file 1: Figure S5) being a suitable MW calibrant due to the absence of sulfate groups that can impact molecular shape [41]. The molecular weight ranges determined by GPC–HPLC (Fig. 7) were similar to those estimated by PAGE analysis (Fig. 8), where both the length of the polysaccharide chains and the distribution of chains of different lengths could be determined [42].

Generally, the *PmHS2* heparosan products from *B. megaterium* displayed a range of molecular weights within two categories: a relatively sharp peak representing MW values greater than 200 kD and a second broader peak encompassing polydisperse products from ~ 10 to 50 kD. GPC data shows that ~ 85% of the shake flask cell pellet heparosan was in the high MW fraction and the remainder was distributed within a broad low MW peak. The primarily high MW cell pellet product identified by GPC is also apparent in lane C of the gel (Fig. 8), which shows a lower smear as well as a poorly separated dark band at the top of the gel, representing the different MW products. A similar profile was observed for the bioreactor cell pellet product’s GPC profile (Fig. 7a) with about half of the product in the low MW fraction, as reflected in the wider distribution of bands in gel lane B of Fig. 8. This corresponds to previous results that found a decrease in MW and increase in product polydispersity of the CPS when moving from shake flask to fed-batch fermentation cultures [32].

The GPC profile of the shake flask supernatant product (Fig. 7c) showed that only 65% of the final product was high MW heparosan and remaining fractions were ~ 25 kD, with some additional lower MW fractions. This is also illustrated in Lane D of Fig. 8, where high MW bands and smears are seen in addition to a series of discrete bands for the fast-moving low MW components at the bottom of the gel. Isolation and ^1^H NMR analysis of these low MW fractions using a 100 kD spin column showed that heparosan in this fraction had a lower degree of *N*-acetylation compared to the high MW fractions. This is an expected result, as heparosan recovered from the cell pellet typically exhibits different characteristics to heparosan recovered from the cell culture supernatant (i.e., higher MW heparosan obtained from the pellet) [1]. For the *E. coli* K5 product (Fig. 7), the MW ranged from ~ 20 to 80 kD [1] similar to heparosan from other hosts such as *B. subtilis* [32]. Relative GPC profiles for the varying amounts of heparosan products in Fig. 7 are also shown (Additional file 1: Figure S6).

## Discussion

In this study we explored the use of the Gram-positive endotoxin-free host organism *B. megaterium* for producing heparosan CPS. Titers of ~ 250 mg/L were achieved in shake flasks and 2.74 g/L in a bioreactor with growth on a minimal medium, using the *PmHS2* gene that polymerizes a heparosan chain from UDP-GlcNAc and UDP-GlcA sugar nucleotide donors. This compares favorably to shake flask heparosan titers of 237.6 mg/L recently reported from *Bacillus subtilis*, using the bifunctional glycosyltransferases *PmHS1* and grown on LB medium. *PmHS2* has previously been shown to be capable of lengthening heparosan chains to different polymerization degrees, leading to a more polydisperse heparosan product [15]. Additionally, *PmHS2* synthesizes heparosan polymers with a much higher average MW compared to *E. coli* K5 heparosan biosynthesis genes that typically produce CPS of 50–80 kD in *E. coli* [1, 15] and other hosts such as *B. subtilis* [32].

The shake flask cell pellet product consisted primarily of high MW heparosan, while products from the shake flask supernatant and bioreactor cell pellet had varying ratios of the high and low MW heparosan. For the shake flask experiments, a greater portion of low MW heparosan was found in the product obtained from the cell culture supernatant, compared to heparosan derived from the cell pellet. The presence of heparosan in the supernatant can be attributed to the shearing action caused by mechanical agitation in the baffled flask over the ~ 48 h growth period, compared to the shorter bioreactor growth times. Overall, the desired product was primarily obtained from the cell pellet, indicating that there may be no well-developed export mechanism for heparosan in *B. megaterium*.

Although *B. megaterium* is a sizeable organism and can potentially accumulate and store greater amounts of intracellular products, a large size also reduces surface-area-to-volume ratio, thereby limiting the capture of nutrients to support the cell’s metabolic needs [43]. This factor, in conjunction with the choice of overexpressed genes, may account for the lower overall product yield when compared to smaller Bacilli like *B. subtilis* [32]. Expansion of our fed-batch growth to a larger volume bioreactor is likely to increase final product titers by permitting longer induction periods before maximum capacity is reached. Additionally, introducing metabolic pathway genes that will drive flux toward heparosan and its precursors can increase titers.

This non-pathogenic host organism provides an alternative source of heparosan, moving away from animal-derived materials and the complex chemical transformation steps involved in reverting HS or heparin to heparosan. Use of this asporogenic *B. megaterium* strain also eliminates the highly energy-consuming sporulation process and offers an improved industrial production strain for biotechnological processes that would otherwise be impaired by possible endospore formation.

## Conclusions

This work provides an alternative and safer method for metabolic engineering of heparosan from a GRAS organism, representing the first instance of heparosan biosynthesis in *B. megaterium.* The structure of the heparosan product was confirmed by LCMS and NMR analyses and presents improved production of this CPS compared to previously reported *B. subtilis* yields using the bifunctional *PmHS1*. Additionally, the higher MW heparosan product described in this study has the added potential for drug delivery applications and use in developing hydrogels and viscoelastic biomaterials with improved performance. Moreover, the low MW heparosan fractions may be useful as a possible starting material for low MW heparin synthesis when combined with downstream enzymatic or chemical modifications [44]. This therefore serves as an additional source of heparosan for a variety of applications, using an inexpensive and readily available carbon source.

## Materials and methods

### Culture media

Three types of media were used for experiments in this study. A growth medium known as Modified Medium [32] consisted of 20 g/L yeast extract, 1.5 g/L MgSO_4_, 50 mM potassium phosphate buffer (pH 7.0), and 50 g/L sucrose as carbon source was used in shake flask experiments. A rich defined medium developed from adapted protocols [36] (known as AMM) consisted of (3.5 g/L KH_2_PO_4_, 5.0 g/L K_2_HPO_4_, 3.5 g/L (NH_4_)_2_HPO_4_, 100 mL of 10× MOPS Mix, 1 mL of 1 M MgSO_4_, 0.1 mL of 1 M CaCl_2_, 1 mL of 0.5 g/L Thiamine HCl, supplemented with 2% (v/v) glucose as a carbon source. 10X MOPS Mix consisted of 83.7 g/L MOPS, 7.2 g/L Tricine, 28 mg/L FeSO_4_·7H_2_O, 29.2 g/L NaCl, 5.1 g/L NH_4_Cl, 1.1 g/L MgCl_2_, 0.5 g/L K_2_SO_4_, 0.2 mL Micronutrient Stock (Micronutrient stock contained 0.2 g/L (NH_4_)_6_Mo_7_O_24_, 1.2 g/L H_3_BO_3_, 0.1 g/L CuSO_4_, 0.8 g/L MnCl_2_, 0.1 g/L ZnSO4). An optimized minimal medium for *B. megaterium* growth, known as M9+ [24], was also used for shake flask experiments and bioreactor fermentations. 1 L of M9+ consisted of 200 mL 5× M9 Salts (Difco, BD), 3 mg/L FeSO_4_, 0.1% casamino acids, 2% (v/v) glucose as a carbon source, 2 mL 1 M MgSO_4_, 100 μL 1 M CaCl_2_, 36 μL 1 M FeSO_4_ and 41.4 μL 1 M MnSO_4_. Final pH of the medium was adjusted to 7.0. Luria-Bertani (LB) medium was used for overnight cell culture growth. The media were supplemented with appropriate antibiotics to sustain the selective pressure on the stable replication of the corresponding plasmids. Tetracycline and chloramphenicol were used at a final concentration of 10 μg/mL and of 4.5 μg/mL respectively for *B. megaterium,* and 80 μg/mL ampicillin was used for *E. coli*. All nutrients and chemicals for medium preparation were from Sigma Chemical Co. (St. Louis, MO).

### Plasmid construction

The commercially available pPT7 and pT7-RNAP shuttle vectors (MoBiTec GmbH) were used for recombinant gene expression in *B. megaterium*). The pT7-RNAP plasmid contains the T7 RNAP gene controlled by the strong *xylA* promoter and the pPT7 plasmid is responsible for the T7 RNAP-dependent expression of the target gene.

The *PmHS2* gene from *Pasteurella multocida*, a dual-action glycosyltransferase, was amplified by a polymerase chain reaction (PCR) using Accuzyme^®^ mix (BIOLINE) according to the manufacturer’s instructions. The PCR product and pPT7 plasmid were digested with restriction enzymes and ligated at the *Nde*I and *Spe*I sites to form a construct known as pPT7_PmHS2. This plasmid was transformed into *E. coli* DH5α by heat shock of chemically competent cells. Several colonies were selected for colony PCR and the correct construct was verified using both double endonuclease digestion and DNA sequencing (Genewiz). The resulting construct was then transformed into *B. megaterium* MS941 alongside the pT7-RNAP plasmid (Fig. 2), by protoplast transformation following previously published protocols [45], to allow for xylose-inducible T7 expression of the target gene.

A negative control strain that was not carrying the *PmHS2* gene was also prepared, creating the pPT7_X expression construct, which was used as a negative control strain for heparosan production (Table 2). Colonies obtained from successful transformations were tested for antibiotic resistance and saved as glycerol stocks, after which their heparosan production potentials were evaluated. The primers, plasmids, and strains used in this study are listed in Table 2. Plasmid DNA was prepared by E.Z.N.A plasmid mini kit (OMEGA) and digested DNA fragments were recovered from agarose gel (Bio-Rad) by E.Z.N.A. gel extraction kit (OMEGA). FastDigest Restriction endonuclease and Rapid DNA ligation kit were purchased from Thermo.

**Table 2:** List of strains, plasmids and primers used for heparosan biosynthesis in *B. megaterium*

Strain/plasmid	Description	Source/references
Strain		
*E. coli* DH5α	General cloning host	Invitrogen
*B. megaterium* MS941	Defined protease deficient mutant of parent strain (DSM319) *ΔnprM*	[23, 46]
Plasmids		
pPT7	T7 promoter, antibiotic resistances against ampicillin (in *E. coli*) and tetracycline (in *B. megaterium*)	MoBiTec GmbH
pT7-RNAP	T7 RNAP gene, *xylA* promoter, antibiotic resistances against ampicillin (in *E. coli*) and chloramphenicol (in *B. megaterium*)	MoBiTec GmbH
pPT7_ PmHS2	pPT7 vector carrying *PmHS2* gene	This study
pPT7_X	pPT7 vector without *PmHS2* gene (negative control strain)	This study

Primer name	Sequence (5′→3′)	Restriction site
PmHS2_F	ccgcgGCTAGCATGAAGAGAAAAAAAGAGATGACTCAAATTCAAATAGC	*Nhe*I
PmHS2_R	ccgcgcACTAGTTATAAAAAATAAAAAGGTAAACAGGGGATAAGGTCAG	*Spe*I
pPT7_seq_F	CCTTTACCTTGTCTACAAACCCC	N/A
pPT7_seq_R	GGTTTGCGCATTCACAGTTCTCC	N/A

### Shake flask and bioreactor growth optimization

Colonies of the engineered *B. megaterium* strains were picked from a streaked 10 μg/mL chloramphenicol and 4.5 μg/mL tetracycline agar plate and used to inoculate 5 mL of LB media in 15 mL curved-bottom culture tubes to grow seed cultures overnight. These were left overnight shaking at 37 °C, 225 rpm, at an angle of ~ 55°, for a maximum of 12 h. These conditions minimized the settling of cells at the bottom of the tube and allowed for consistency with expression. For shake flask cultivations, ~ 5 mL of this seed culture was added to 50 mL of each type of growth medium, in a 250 mL Erlenmeyer flask so that the initial optical density at 600 nm (OD_600_) was ~ 0.05. Growth was tested in a modified medium with sucrose as carbon source [32], AMM—a rich defined medium developed from modified protocols [47], and M9+ [24]—a minimal media optimized for *B. megaterium* growth, to identify which was optimal for cell growth and heparosan production. The volume of cell culture was no more than 1/5th the volume of the shake flask in order to ensure proper aeration during growth.

The cell culture was incubated in a rotary air shaker (New Brunswick Scientific Innova 44R) at 37 °C, 225 rpm. Samples were removed from the shake flasks occasionally to plot growth curves and assess glucose uptake by the cells. When OD_600_ was measured to be between 0.33 and 0.50 (after ~ 4–6 h of growth), recombinant expression of the *PmHS2* gene under transcriptional control of the xylose promoter was induced by the addition of 20 g/L xylose. The cells were allowed to grow for a further 48 h until late exponential phase. Cells were separated from the growth medium by centrifugation at 4 °C (5500×*g* for 1 h). The cell pellet was stored at − 20 °C for further analysis. The cell culture supernatant was filtered using a 0.45 μm Corning^®^ bottle-top vacuum filter to remove solid particulates, then concentrated to ~ 10 mL by tangential flow filtration using a Vivaflow 200 cassette (Sartorius) with exclusion size of 10 kDa.

Fed-batch fermentation was carried out in a 1.5 L DASGIP fermenter (Eppendorf) with 1 L of 2% glucose M9+ medium. A 50 mL seed culture was grown overnight in LB medium at 37 °C, 225 rpm then spun down to remove the growth medium. The cell pellet was re-suspended in 5 mL M9+ and this inoculum was added to the bioreactor, such that the starting OD_600_ was ~ 0.1. Fermentation was carried out at 37 °C and pH was maintained at 7.0 by addition of 15% NH_4_OH (Millipore Sigma) as needed. Agitation rate was maintained at 500 rpm initially and increased to 600 rpm at the end of the log phase, to maintain the dissolved oxygen value at 30%. 20 g/L of xylose was added at OD_600_ 0.35–0.50 to initiate induction and 40% glucose solution was fed to maintain a 0.4 h^−1^ growth rate. 1 mL fermentation broth aliquots were removed from the bioreactor periodically to track OD_600_ and sugar consumption. Aliquots were centrifuged for 5 min at 8000×*g* and xylose and glucose consumption were measured using 200 µL of supernatant by HPLC analysis, using Agilent 1200 series HPLC equipped with a Zorbax Carbohydrate column (5 µm, 4.6 × 150 mm) and a refractive index detector. The mobile phase was a 75% acetonitrile and 25% water mixture at a flow rate of 2 mL/min. Sugar concentrations were determined using authentic standards.

A 50 mL sample was removed at the end of fermentation when the bioreactor reached its maximum capacity (~ 20 h after induction) and the supernatant and pellet were purified and processed separately for heparosan analysis and quantification by LCMS.

### Molecular weight analysis

GPC-HPLC was used to determine the molecular weight and polydispersity of the heparosan samples [41]. Two analytical columns: TSK G4000 SWXL 7.8 mm × 30 cm, 8 μm in series with TSK G3000SWXL 7.8 mm × 30 cm, 5 μm (Tosoh Corporation, Tokyo, Japan), were protected by a guard column TSK SWXL 6 mm × 4 cm, 7 μm diameter. These columns were connected to an HPLC system comprising a Shimadzu RID-10A refractive index detector, LC-10Ai pump, and CBM-20A controller (Shimadzu, Kyoto, Japan). The mobile phase was 0.1 M ammonium acetate with 0.02% (w/v) sodium azide. An Eppendorf column heater (Eppendorf, Hamburg, Germany) was used to maintain the columns and refractive index detector at 30 °C. The sample injection volume was 20 μL with concentrations of ~ 5 mg/mL and the flow rate was 0.6 mL/min. For molecular weight determination, a range of dextran standard calibrants was used.

The purified cell pellet and cell culture supernatant heparosan products were also analyzed using polyacrylamide-gel electrophoresis (PAGE) with a 15% total acrylamide resolving gel, as previously described [16, 41, 48]. To visualize the ion front during electrophoresis, a phenol red dye prepared in 50% (w/v) sucrose was added to ~ 10 µg of each sample. The standard consisted of a mixture of enzymatically prepared heparin oligosaccharides of known molecular weight from bovine lung heparin [49]. The gel was fixed with Alcian blue dye and digitized with a ChemiDoc Molecular Imager and Image Lab Software (Bio-Rad).

### Heparosan purification

The concentrated fermentation supernatant was digested using 1 mg/mL DNAse (Sigma) for 1 h at 37 °C, then 2.5 mg/mL Actinase E at 56 °C for 10 h. It was then further concentrated using a pre-rinsed 3 kD Amicon Ultra-15 centrifugal filter unit at 4000×*g*. The retentate containing heparosan was desalted on the spin column by repeated resuspension in deionized water to remove residual salts and small peptides, followed by centrifugation and overnight lyophilization. Samples were then re-dissolved in a binding buffer (20 mM sodium acetate, pH 5) and mixed with DEAE (diethylaminoethyl) Sepharose fast flow resin (GE Lifesciences) that was washed and pre-equilibrated with the same buffer in a 25 mL polypropylene gravity flow column (BioRad). After overnight incubation with shaking at room temperature, the column was washed with 4 column volumes of binding buffer, followed by a series of elutions of increasing salt concentrations (100 mM, 200 mM, 500 mM and 1 M NaCl in 20 mM sodium acetate, pH 5) to recover the bound heparosan. These fractions were desalted using 3.5 kD molecular weight cut-off Slide-A-Lyzer™ Dialysis Cassettes (ThermoFisher Scientific) in deionized water, then lyophilized for further analysis. The cell pellet was lysed using a CelLytic™ B Plus Kit (Sigma) according to the user protocol. Additional cell disruption was carried out by autoclaving for 15 min on the liquid cycle. The lysate was centrifuged at 12,000×*g* for 1 h the supernatant was purified as previously described for the cell culture supernatant.

### NMR analysis

The purified CPS from the supernatant was analyzed by one-dimensional ^1^H nuclear magnetic resonance (NMR) [50]. NMR experiments were performed on a Bruker Advance II 600 MHz spectrometer (Bruker Bio Spin, Billerica, MA) with Topsin 2.1.6 software (Bruker). Samples were dissolved in 0.5 mL D_2_O (99.996%, Sigma Chemical Company) and freeze-dried repeatedly to remove the exchangeable protons. The samples were re-dissolved in 0.4 mL D_2_O and transferred to NMR microtubes [outside diameter, 5 mm, Norell (Norell, Landisville, NJ)]. As previously described [36], the conditions for one-dimensional ^1^H NMR spectra were as follows: wobble sweep width of 12.3 kHz, acquisition time of 2.66 s, and relaxation delay of 8.00 s; temperature was 298 K. NMR heparosan standard spectral data was used to confirm peak assignments and assess product purity.

### Heparosan quantification using liquid chromatography mass spectrometry (LCMS)

Complete depolymerization of heparosan products was performed using recombinant heparin lyase I, II and III, that were expressed and purified as previously described [51–53]. A range of heparosan amounts (within the limit of detection of the LCMS instrument) was spiked into the cell pellet supernatant of the negative control strain to develop a standard curve for heparosan quantification; samples were prepared in triplicate. Purified heparosan samples from both the supernatant and cell pellet, as well as the heparosan standard, were mixed with 150 μL digestion buffer (50 mM ammonium acetate, pH 7.5). The heparin lyases (~ 20 mU) were added and the reaction mixtures were incubated at 37 °C overnight for complete depolymerization.

The digested solutions were filtered through a 3 kD column and the filtrates were collected and lyophilized. The freeze-dried samples containing heparosan disaccharides or heparosan disaccharide standards were added to 10 μL of 0.1 M AMAC solution in acetic acid (AcOH)/DMSO (3:17, v/v) and mixed by vortexing for 5 min. Next, 10 μL of 1 M sodium cyanoborohydride was added to the reaction mixture and incubated at 45 °C for 1 h. After the AMAC-labeling reaction, the samples were centrifuged at 13,000×*g* for 10 min and the supernatants were recovered. Liquid chromatography–mass spectrometry (LCMS) analyses were carried out on the AMAC-tagged disaccharide using an Agilent 1200 LC/MSD instrument (Agilent Technologies, Inc. Wilmington, DE) according to published protocols [37]. For more sensitive MS analysis, a Thermo Electron Finnigan TSQ Quantum Ultra was used on AMAC labeled samples as described elsewhere [54]. The data acquired was analyzed using Thermo Xcalibur software, and disaccharides were quantified using peak integration and an external standard.

## Additional file

**Additional file 1: Figure S1.** Standard curve used for heparosan quantification by LCMS disaccharide analysis. **Figure S2.** Shake flask heparosan titers from various combinations of induction OD_600_ values (0.20, 0.33, 0.50, 0.75), lengths of induction periods (14 h, 24 h or 48 h), and induction temperatures (30 °C and 37 °C) in M9+ medium. **Figure S3.** (A) Sugar consumption profile for DASGIP bioreactor growth of heparosan-producing *B. megaterium* strain over 24 h period. (B) Standard curve for HPLC quantification of glucose and xylose in fermentation broth. **Figure S4.** ^1^H NMR spectrum of heparosan product from *E. coli* K5 prepared as previously described (17). **Figure S5.** Dextran standards used as a MW calibrant for gel permeation chromatography–high performance liquid chromatography (GPC–HPLC) measurement of the relative molecular mass properties of *B. megaterium* heparosan products. **Figure S6.** Overlaid molecular weight profiles of various heparosan products measured by GPC-HPLC in Figure 7. Molecular weights were determined using dextran calibration standards as indicated by the labeled arrows.

## Data Availability

The datasets used and/or analyzed during this study are included in this published article [and its additional file] or are available from the corresponding author on reasonable request.

## Abbreviations

AMAC: 2 aminoacridone
CPS: capsular polysaccharide
GAG: glycosaminoglycan
GRAS: generally regarded as safe
GPC: gel permeation chromatography
HPLC: high performance liquid chromatography
LB: Luria–Bertani
LC–MS: liquid chromatography–mass spectrometry
LMW: low molecular weight
MRM: multiple reaction monitoring
MW: molecular weight
NMR: nuclear magnetic resonance
OD_600_: optical density at 600 nm
Tris-HCl: tris(hydroxymethyl)aminomethane hydrochloride
