# Supplementary figures and images for: Metabolic engineering of Bacillus megaterium for heparosan biosynthesis using Pasteurella multocida heparosan synthase, PmHS2
Source: Microb Cell Fact. 2019 Aug 12;18:132. doi: 10.1186/s12934-019-1187-9 (PMC6691538; doi:10.1186/s12934-019-1187-9)

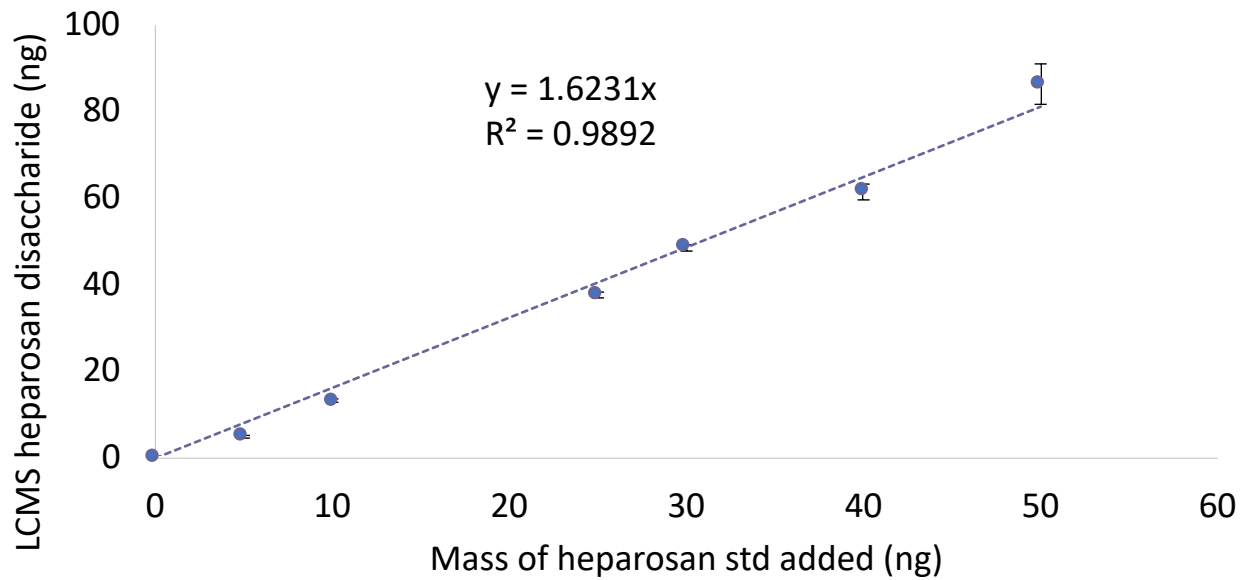

Supplement: Supplementary file 1 — Additional file 1: Figure S1. Standard curve used for heparosan quantification by LCMS disaccharide analysis. Figure S2. Shake flask heparosan titers from various combinations of induction OD600 values (0.20, 0.33, 0.50, 0.75), lengths of induction periods (14 h, 24 h or 48 h), and induction temperatures (30 °C and 37 °C) in M9+ medium. Figure S3. (A) Sugar consumption profile for DASGIP bioreactor growth of heparosan-producing B. megaterium strain over 24 h period. (B) Standard curve for HPLC quantification of glucose and xylose in fermentation broth. Figure S4. 1H NMR spectrum of heparosan product from E. coli K5 prepared as previously described (17). Figure S5. Dextran standards used as a MW calibrant for gel permeation chromatography–high performance liquid chromatography (GPC–HPLC) measurement of the relative molecular mass properties of B. megaterium heparosan products. Figure S6. Overlaid molecular weight profiles of various heparosan products measured by GPC-HPLC in Figure 7. Molecular weights were determined using dextran calibration standards as indicated by the labeled arrows. [file 12934_2019_1187_MOESM1_ESM.zip › Additional File 1, Figure S1.pdf]

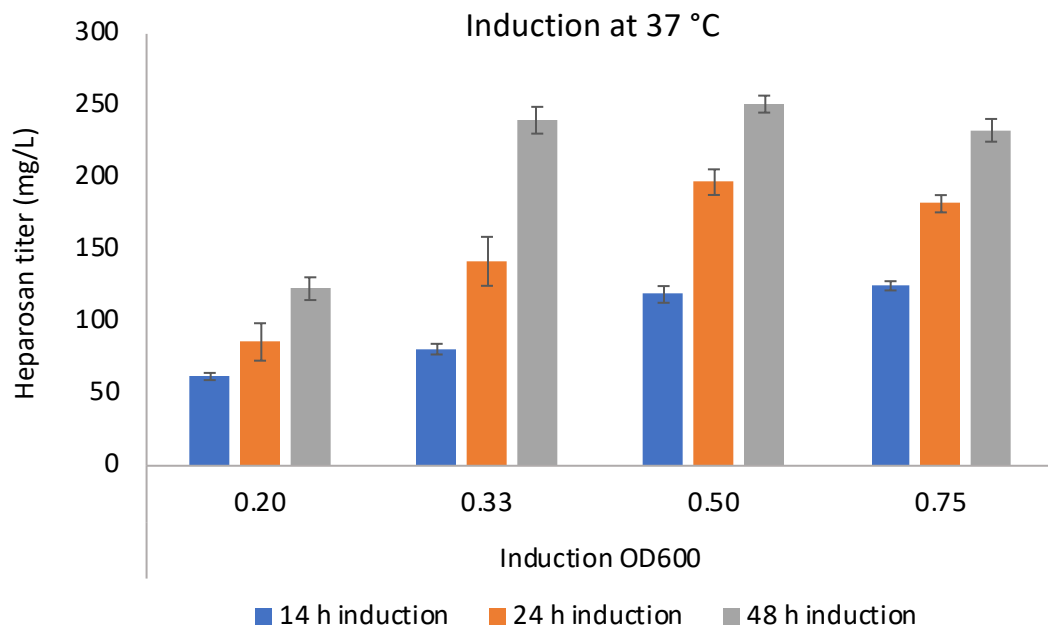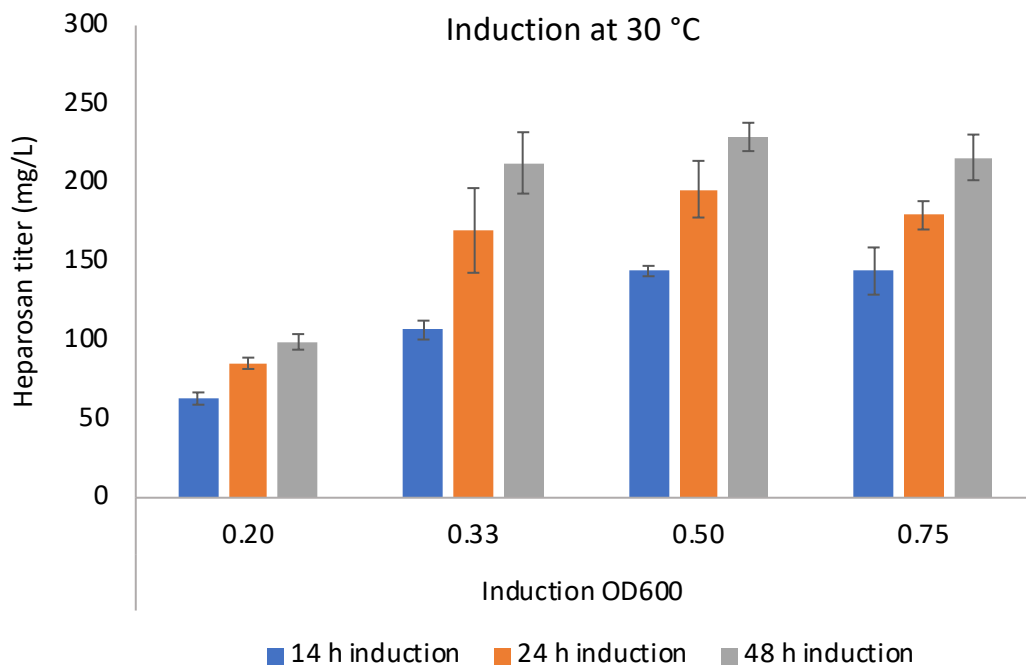

Supplement: Supplementary file 1 — Additional file 1: Figure S1. Standard curve used for heparosan quantification by LCMS disaccharide analysis. Figure S2. Shake flask heparosan titers from various combinations of induction OD600 values (0.20, 0.33, 0.50, 0.75), lengths of induction periods (14 h, 24 h or 48 h), and induction temperatures (30 °C and 37 °C) in M9+ medium. Figure S3. (A) Sugar consumption profile for DASGIP bioreactor growth of heparosan-producing B. megaterium strain over 24 h period. (B) Standard curve for HPLC quantification of glucose and xylose in fermentation broth. Figure S4. 1H NMR spectrum of heparosan product from E. coli K5 prepared as previously described (17). Figure S5. Dextran standards used as a MW calibrant for gel permeation chromatography–high performance liquid chromatography (GPC–HPLC) measurement of the relative molecular mass properties of B. megaterium heparosan products. Figure S6. Overlaid molecular weight profiles of various heparosan products measured by GPC-HPLC in Figure 7. Molecular weights were determined using dextran calibration standards as indicated by the labeled arrows. [file 12934_2019_1187_MOESM1_ESM.zip › Additional FIle 1, Figure S2.pdf]

A

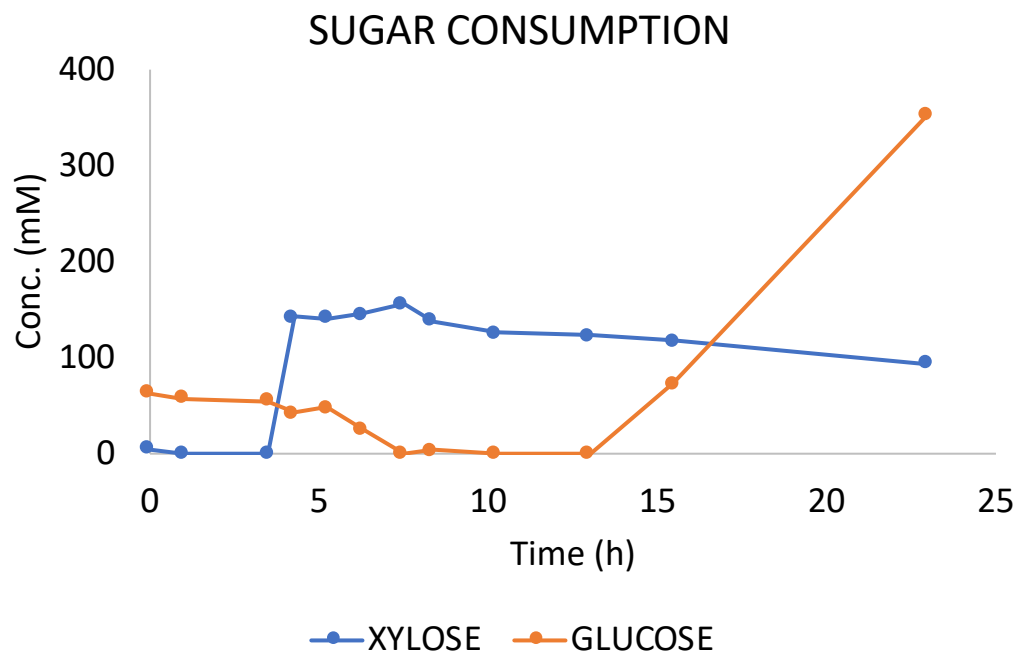

B

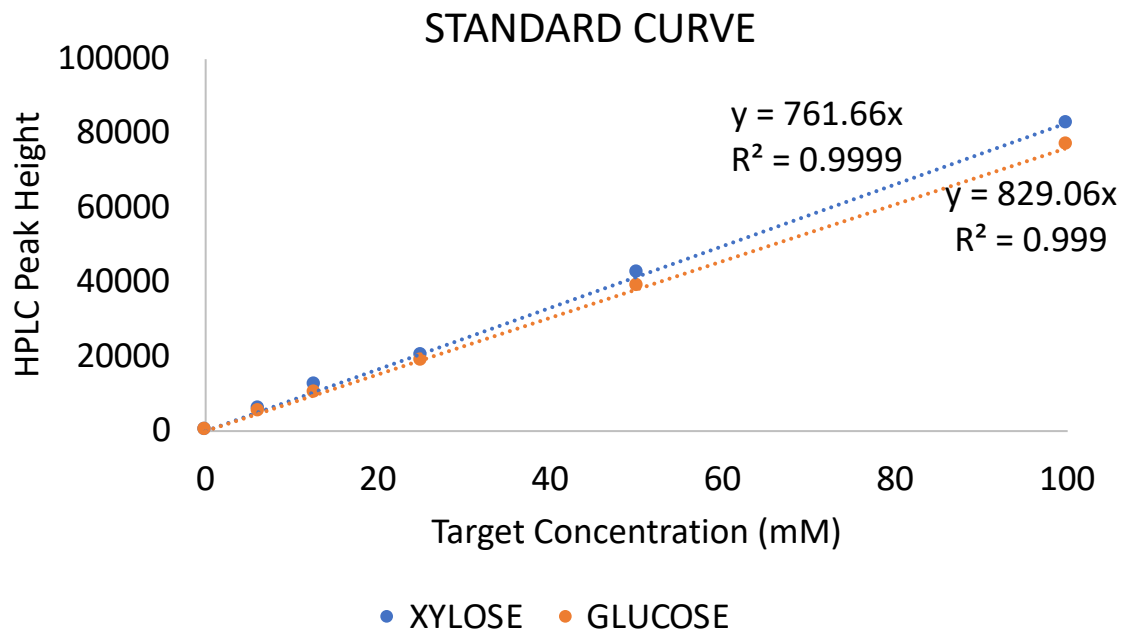

Supplement: Supplementary file 1 — Additional file 1: Figure S1. Standard curve used for heparosan quantification by LCMS disaccharide analysis. Figure S2. Shake flask heparosan titers from various combinations of induction OD600 values (0.20, 0.33, 0.50, 0.75), lengths of induction periods (14 h, 24 h or 48 h), and induction temperatures (30 °C and 37 °C) in M9+ medium. Figure S3. (A) Sugar consumption profile for DASGIP bioreactor growth of heparosan-producing B. megaterium strain over 24 h period. (B) Standard curve for HPLC quantification of glucose and xylose in fermentation broth. Figure S4. 1H NMR spectrum of heparosan product from E. coli K5 prepared as previously described (17). Figure S5. Dextran standards used as a MW calibrant for gel permeation chromatography–high performance liquid chromatography (GPC–HPLC) measurement of the relative molecular mass properties of B. megaterium heparosan products. Figure S6. Overlaid molecular weight profiles of various heparosan products measured by GPC-HPLC in Figure 7. Molecular weights were determined using dextran calibration standards as indicated by the labeled arrows. [file 12934_2019_1187_MOESM1_ESM.zip › Additional File 1, Figure S3.pdf]

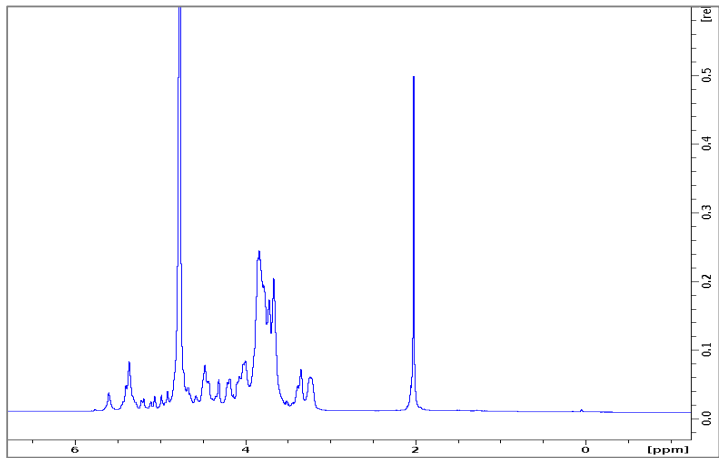

Supplement: Supplementary file 1 — Additional file 1: Figure S1. Standard curve used for heparosan quantification by LCMS disaccharide analysis. Figure S2. Shake flask heparosan titers from various combinations of induction OD600 values (0.20, 0.33, 0.50, 0.75), lengths of induction periods (14 h, 24 h or 48 h), and induction temperatures (30 °C and 37 °C) in M9+ medium. Figure S3. (A) Sugar consumption profile for DASGIP bioreactor growth of heparosan-producing B. megaterium strain over 24 h period. (B) Standard curve for HPLC quantification of glucose and xylose in fermentation broth. Figure S4. 1H NMR spectrum of heparosan product from E. coli K5 prepared as previously described (17). Figure S5. Dextran standards used as a MW calibrant for gel permeation chromatography–high performance liquid chromatography (GPC–HPLC) measurement of the relative molecular mass properties of B. megaterium heparosan products. Figure S6. Overlaid molecular weight profiles of various heparosan products measured by GPC-HPLC in Figure 7. Molecular weights were determined using dextran calibration standards as indicated by the labeled arrows. [file 12934_2019_1187_MOESM1_ESM.zip › Additional File 1, Figure S4.pdf]

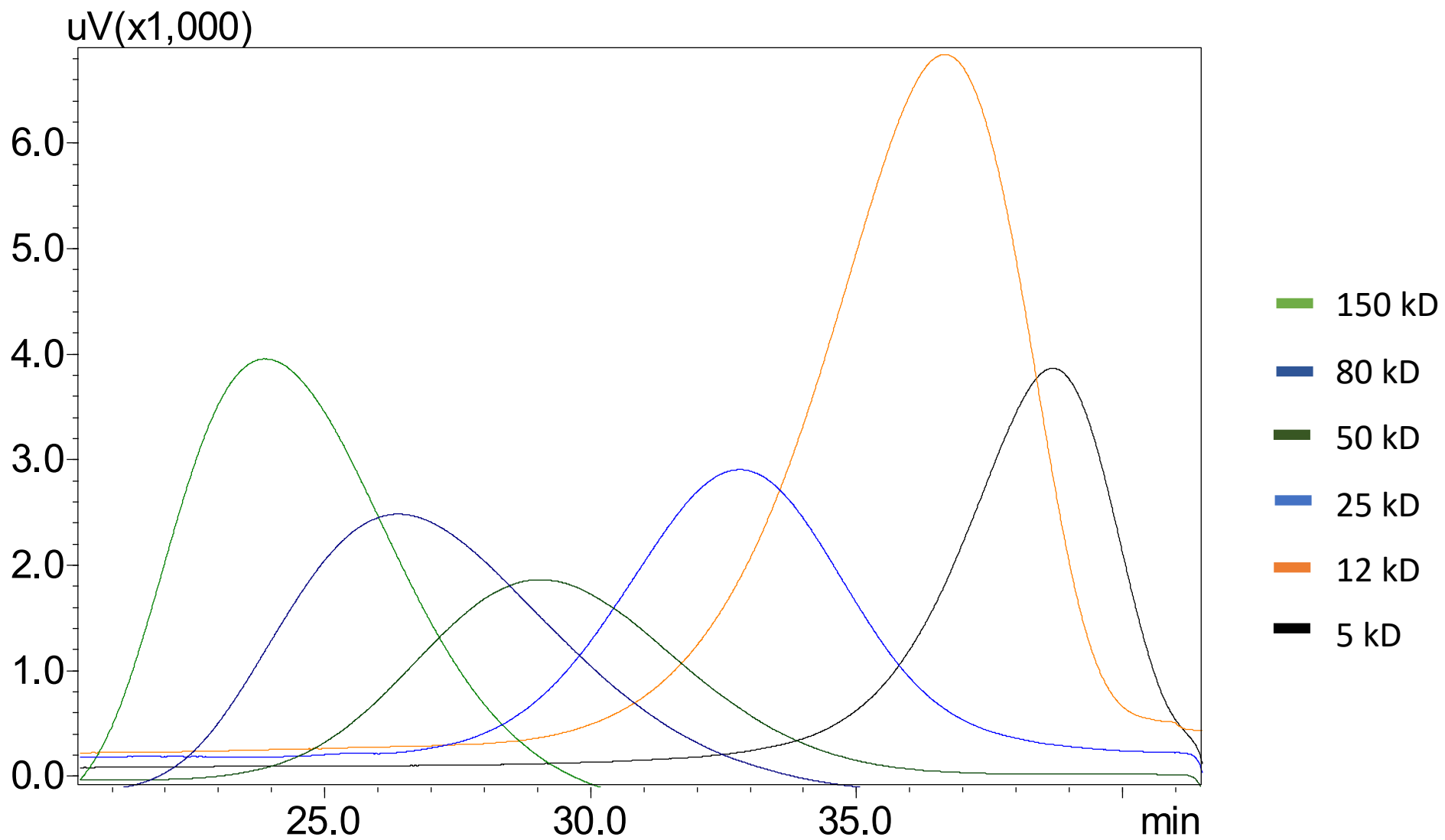

Supplement: Supplementary file 1 — Additional file 1: Figure S1. Standard curve used for heparosan quantification by LCMS disaccharide analysis. Figure S2. Shake flask heparosan titers from various combinations of induction OD600 values (0.20, 0.33, 0.50, 0.75), lengths of induction periods (14 h, 24 h or 48 h), and induction temperatures (30 °C and 37 °C) in M9+ medium. Figure S3. (A) Sugar consumption profile for DASGIP bioreactor growth of heparosan-producing B. megaterium strain over 24 h period. (B) Standard curve for HPLC quantification of glucose and xylose in fermentation broth. Figure S4. 1H NMR spectrum of heparosan product from E. coli K5 prepared as previously described (17). Figure S5. Dextran standards used as a MW calibrant for gel permeation chromatography–high performance liquid chromatography (GPC–HPLC) measurement of the relative molecular mass properties of B. megaterium heparosan products. Figure S6. Overlaid molecular weight profiles of various heparosan products measured by GPC-HPLC in Figure 7. Molecular weights were determined using dextran calibration standards as indicated by the labeled arrows. [file 12934_2019_1187_MOESM1_ESM.zip › Additional File 1, Figure S5.pdf]

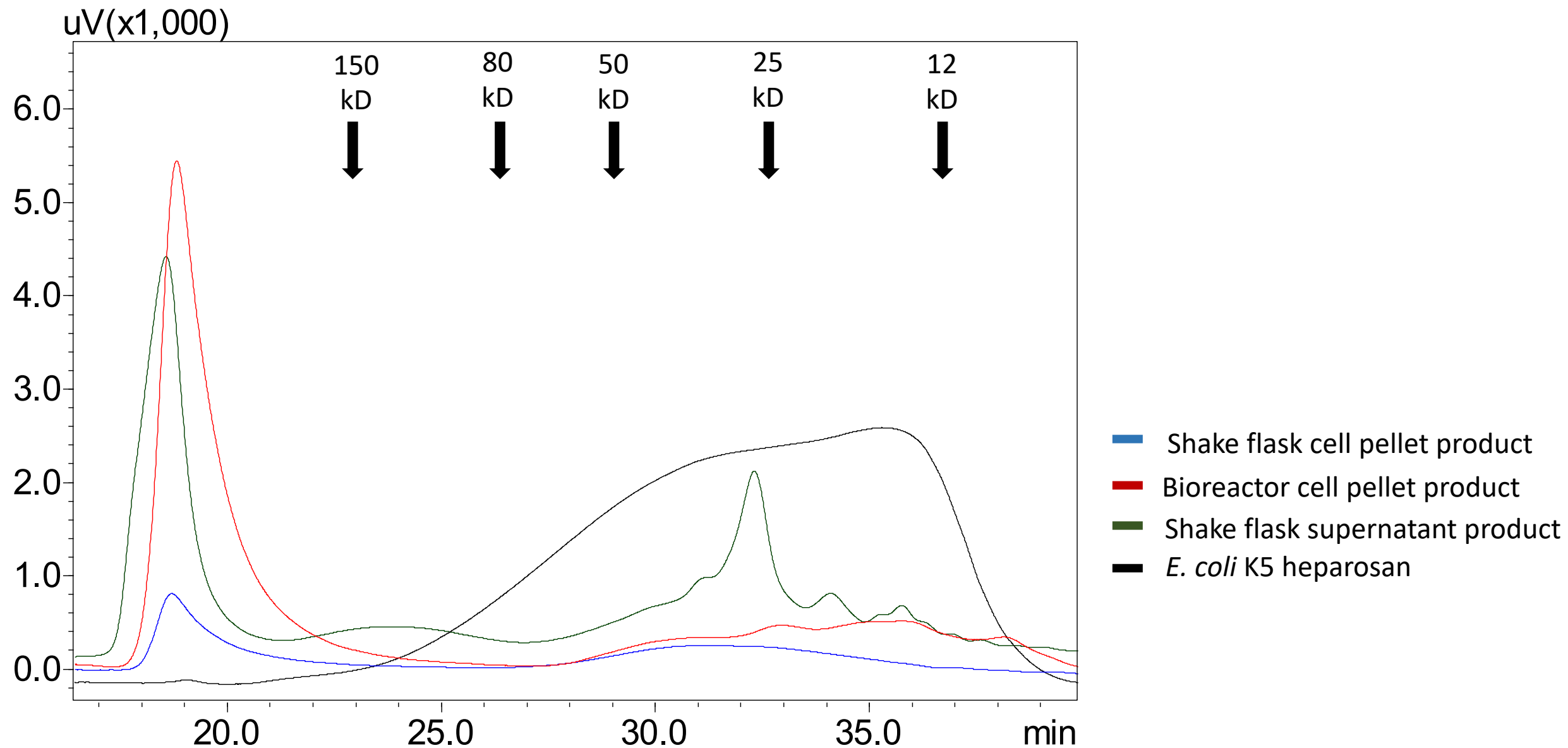

Supplement: Supplementary file 1 — Additional file 1: Figure S1. Standard curve used for heparosan quantification by LCMS disaccharide analysis. Figure S2. Shake flask heparosan titers from various combinations of induction OD600 values (0.20, 0.33, 0.50, 0.75), lengths of induction periods (14 h, 24 h or 48 h), and induction temperatures (30 °C and 37 °C) in M9+ medium. Figure S3. (A) Sugar consumption profile for DASGIP bioreactor growth of heparosan-producing B. megaterium strain over 24 h period. (B) Standard curve for HPLC quantification of glucose and xylose in fermentation broth. Figure S4. 1H NMR spectrum of heparosan product from E. coli K5 prepared as previously described (17). Figure S5. Dextran standards used as a MW calibrant for gel permeation chromatography–high performance liquid chromatography (GPC–HPLC) measurement of the relative molecular mass properties of B. megaterium heparosan products. Figure S6. Overlaid molecular weight profiles of various heparosan products measured by GPC-HPLC in Figure 7. Molecular weights were determined using dextran calibration standards as indicated by the labeled arrows. [file 12934_2019_1187_MOESM1_ESM.zip › Additional File 1, Figure S6.pdf]
